# The effect of colchicine on cholesterol crystal formation, expansion and morphology: a potential mechanism in atherosclerosis

**DOI:** 10.3389/fcvm.2024.1345521

**Published:** 2024-02-16

**Authors:** Zain Ul Abideen, Dorothy R. Pathak, Rand Sabanci, Megan Manu, George S. Abela

**Affiliations:** ^1^ Department of Medicine, Division of Cardiology, Michigan State University, East Lansing, MI, United States; ^2^ Department of Epidemiology and Biostatistics, Michigan State University, East Lansing, MI, United States; ^3^ College of Human Medicine, Michigan State University, East Lansing, MI, United States; ^4^ Department of Physiology, Division of Pathology, Michigan State University, East Lansing, MI, United States

**Keywords:** cholesterol crystals, atherosclerosis, colchicine, plaque rupture, cardiovascular events

## Abstract

**Background:**

Inflammation is pivotal to the progression of atherosclerosis. Cholesterol crystals (CCs) that grow and enlarge within the plaque core can cause plaque rupture and trigger inflammation as they deposit into the atherosclerotic bed. Thus, agents that affect CC formation, expansion, and morphology may reduce cardiovascular (CV) risk independent of lipid-lowering and anti-inflammatory therapy.

**Objective:**

Because colchicine is highly concentrated in leukocytes that can enter the atherosclerotic plaque core, we tested its effect on the formation and growth of CCs in bench experiments to determine whether it may have direct effects on CCs, independent of its known anti-inflammatory actions.

**Method:**

Different dosages of colchicine mixed with cholesterol (0.05–5 mg/ml/g of cholesterol) were used to influence the formation CCs and volume expansion *in vitro*. These were compared to control samples with cholesterol in ddH_2_O without colchicine. In an *ex vivo* study, fresh atherosclerotic human plaques were incubated with and without colchicine in a water bath at 37°C for 48 h to assess the impact of colchicine on CC morphology. Scanning electron microscopy (SEM) was utilized to analyze CC morphology in samples from the various treatment groups.

**Results:**

The addition of colchicine to cholesterol caused a substantial dose-dependent reduction in volume (*p* < 0.05). Pairwise comparisons of volume reduction, showed a significant reduction in volume at 5 mg/ml/g when compared to control (*p* < 0.02) but the calculated Cohen's *d* effect size was large for five of the six pairwise comparisons. By SEM, CCs from both *in vitro* and *ex vivo* samples treated with colchicine had evidence of dissolution and changes in their morphology as evidenced by the loss of their sharp edges. In contrast, CCs in untreated specimens retained their typical geometric structure.

**Conclusions:**

Colchicine can reduce CC formation and expansion and alter CC morphology. These previously unappreciated effects of colchicine may contribute to its clinical benefit in patients with CV disease independent of its anti-inflammatory effects.

## 1 Introduction

Inflammation is fundamental to the development of atherosclerosis and plaque destabilization (1). Recent evidence suggests that cholesterol crystals (CCs) that form within the plaque core can cause plaque rupture as they enlarge and trigger inflammation when they are released into the atherosclerotic bed. Like uric acid crystals, CCs have been demonstrated to activate NLRP3 inflammasomes leading to IL-1β and then IL-6 and C-reactive protein (2–4). Furthermore, as CCs develop and enlarge, they develop sharp tips that can directly perforate the atherosclerotic plaque and trigger atherothrombosis independent of inflammation-induced disruption of the plaque structure (5, 6). Historically, colchicine has been used to treat gout and other inflammatory conditions (7, 8). However, recently it has been shown to significantly reduce recurrent cardiovascular events (CVEs) in high-risk patients (9). Given these findings, we proposed to test the effect of colchicine on CCs to evaluate a role other than anti-inflammation.

The actions of CCs can be interrupted by lowering the available free cholesterol within the plaque by a variety of lipid-lowering approaches but that requires long-term therapy (10). Of interest is the observation that statins and aspirin may directly dissolve CCs and alter their morphology (11–14). Colchicine has been shown to be highly concentrated within leukocytes that traverse the plaque bed and become entrapped in the plaque core. Thus, in contrast to statins and aspirin, it is likely that colchicine can accumulate within atherosclerotic plaque (15, 16). Thus, we evaluated the ability of colchicine to alter CC formation, expansion, and morphology in both *in vitro* and *ex vivo* experiments to determine whether it may have actions that could reduce the risk of atherosclerosis independent of its direct anti-inflammatory effects.

## 2 Methods

### 2.1 Effect of colchicine on cholesterol crystallization *in vitro*

Purified cholesterol powder [5-cholesten-3β-ol; 3β-hydroxy-5-cholestene (C27H46O): molecular weight = 386.7; 95%–98% pure, Sigma, St. Louis, MO, USA] was melted in 10 ml graduated cylinders (Pyrex VISTA, Corning Inc., Corning, NY, USA) using a heating gun (HAG 1,400-U, GAR-TEC, Baden, Germany), and volume expansion was measured as previously described (13). Temperature achieved was at the melting point of cholesterol (147°C). The meniscus level of liquid cholesterol (V1) obtained upon melting was noted. The cylinder was then allowed to cool for 10 min at room temperature and maximal peak of CCs formed (V2) was noted. The peak volume expansion (ΔVE) was calculated by subtracting V1 from V2.

Varying doses of colchicine (C_22_H_25_NO_6_, >95%, Sigma-Aldrich Co. St. Louis, MO, USA) mixed with cholesterol powder and water (0.05–5 mg/ml/g of cholesterol) were melted, and ΔVE was measured as mentioned above and compared to control samples without colchicine. The experiment was repeated six times each and the results were averaged. The crystals were then examined by scanning electron microscopy (SEM). The morphology of CCs formed with colchicine was compared to CC controls without colchicine.

The experimental doses of colchicine employed were designed to be analogous to the exposure levels commonly encountered by arterial plaques in humans. This equivalence was derived from the analysis of colchicine concentrations in the human serum following a single oral administration of 0.5 mg, achieving a maximum serum concentration (Cmax) of 2.1 ng/ml (17). Given that disrupted plaques contain an average of 30 mg/g of free cholesterol and considering a cardiac output of 5 L/min, it was determined that 0.01 mg of colchicine interfaces with free cholesterol at a rate of 30 mg/min. Higher colchicine doses were administered in the experiments, reflecting concentrations up to 600 times greater found in leukocytes that are equivalent to 6.3 mg in contact with 30 mg/min of free cholesterol (16–18). Although the serum levels of colchicine in humans are markedly lower than what was used in the *in vitro* test tube study, the doses used were designed to provide the visual measurements needed for the *in vitro* experiment. Moreover, another study showed that low doses of colchicine (0.1, 1, and 2 mg) all had a comparable effect on ΔVE (19).

### 2.2 Effect of colchicine on cholesterol crystals in human atherosclerotic plaques

Three fresh human carotid plaques were obtained from endarterectomy procedures and cut into equal halves and incubated in a water bath for 48 h at 37°C. One half was incubated in ddH_2_O with colchicine (5 mg/ml), and the other half was incubated in ddH_2_O alone. Plaque specimens were de-identified and were consistent with Sparrow Hospital and Michigan State University IRB approval (# 0518-exempt and no consent was required). Treated plaques were then prepared for SEM and crystal morphology analyzed in samples from the treatment groups of atherosclerotic human plaques incubated with and without colchicine. Two individuals concurred on the SEM findings and a third individual adjudicated if there was disagreement.

### 2.3 Scanning electron microscopy

Atherosclerotic plaques were fixed overnight in buffered 4% glutaraldehyde and then cut into 5-mm segments and air dried as previously described (6). All samples were then mounted on stubs and gold coated in an EMSCOPE SC500 sputter coater (UK), and the surface was examined for CCs using a JEOL scanning electron microscope (Model JSM-6610LV, JEOL., Tokyo, Japan). Also, control and colchicine samples treated *in vitro* were collected and scanned for their morphology.

During SEM examination, crystals were evaluated for elemental composition using energy-dispersive x-ray spectroscopy (EDS) (AZtec system, Oxford Instruments, High Wycombe, Bucks, England). CCs analyzed with EDS were used to confirm the chemical composition corresponding to crystal shapes as previously reported (13).

### 2.4 Statistical analysis

All statistical analyses were performed using SAS version 9.4 (SAS Inc. Cary, NC, USA). Data were the difference between V2 and V1 (ΔVE). One-way ANOVA with Tukey–Kramer multiple-comparison post-tests and non-parametric equivalent, and the Kruskal–Wallis test followed by the Dwass–Steel–Critchlow–Fligner (DSCF) multiple-comparisons test were performed to compare peak volume expansions at various doses of colchicine. The results were the same for the two approaches; therefore, only the parametric comparisons are reported. We also calculated Cohen's *d* effect size (ES) for all pairwise comparisons because of the small sample size. In addition, simple linear regression of ΔVE on the colchicine dose was performed without and with control value (0 colchicine) in the model. Results are reported as means ± SD, and *p* *< *0.05 is used to report statistical significance in all tests.

## 3 Results

### 3.1 Effect of colchicine on cholesterol crystallization *in vitro*

**Figure 1:**
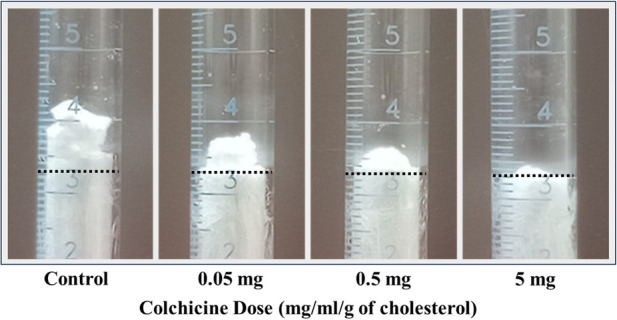
Example of dose-related effect of colchicine. The dotted line is the site of the original meniscus, which is almost equal for all the melted 3 g of cholesterol used.

**Figure 2:**
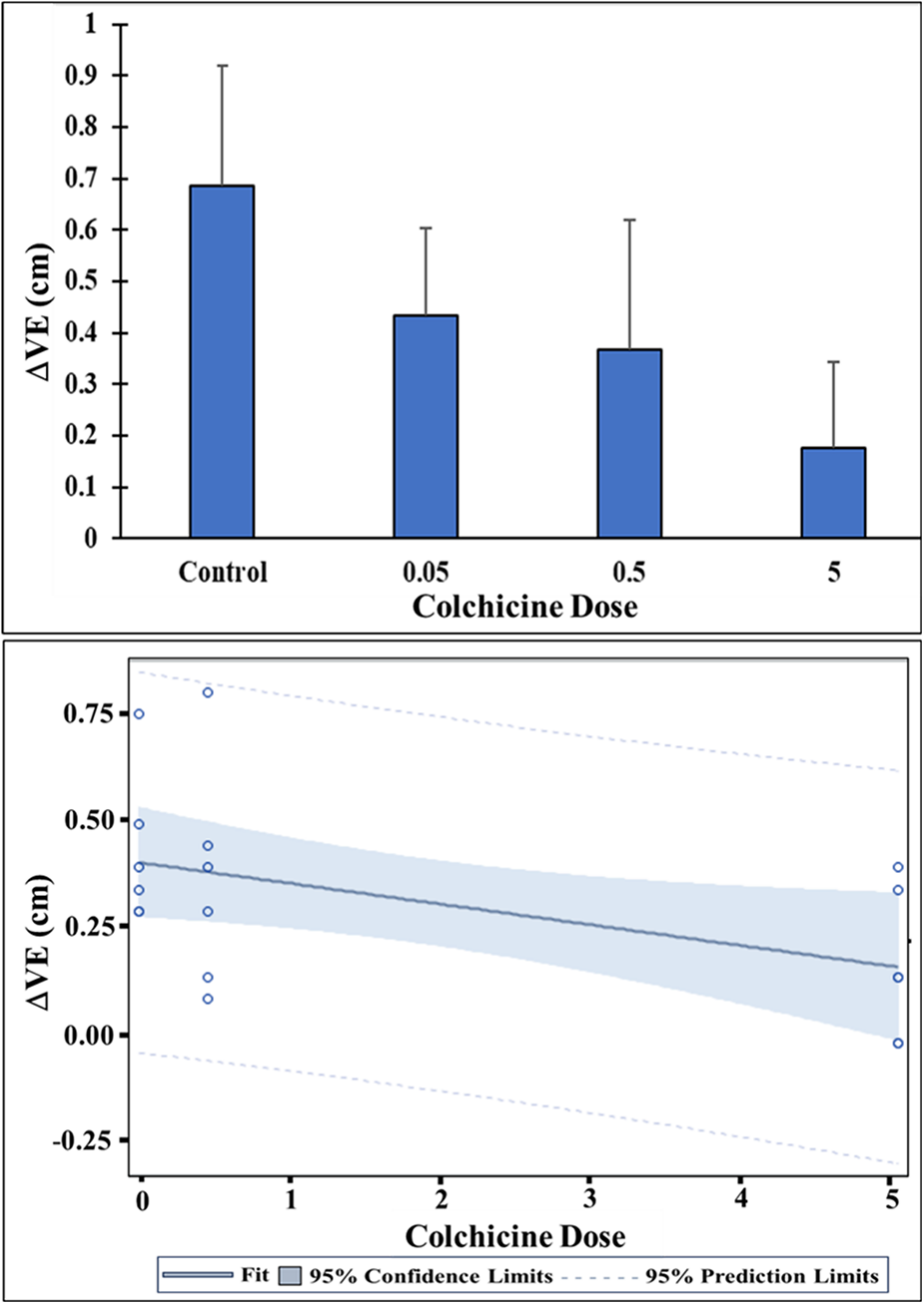
Change in cholesterol volume expansion (ΔVE) with and without colchicine. Control ΔVE differed significantly from ΔVE for the 5 mg/ml/g colchicine dose, *p* < 0.02. Colchicine treatment pairwise group comparisons were not significantly different possibly related to sample size. However, there is a dose-related decline in ΔVE with increasing levels of colchicine. Linear regression of ΔVE on the colchicine dose showed a significant decreasing trend in ΔVE with increasing dose of colchicine, at *p* < 0.05.

**Figure 3:**
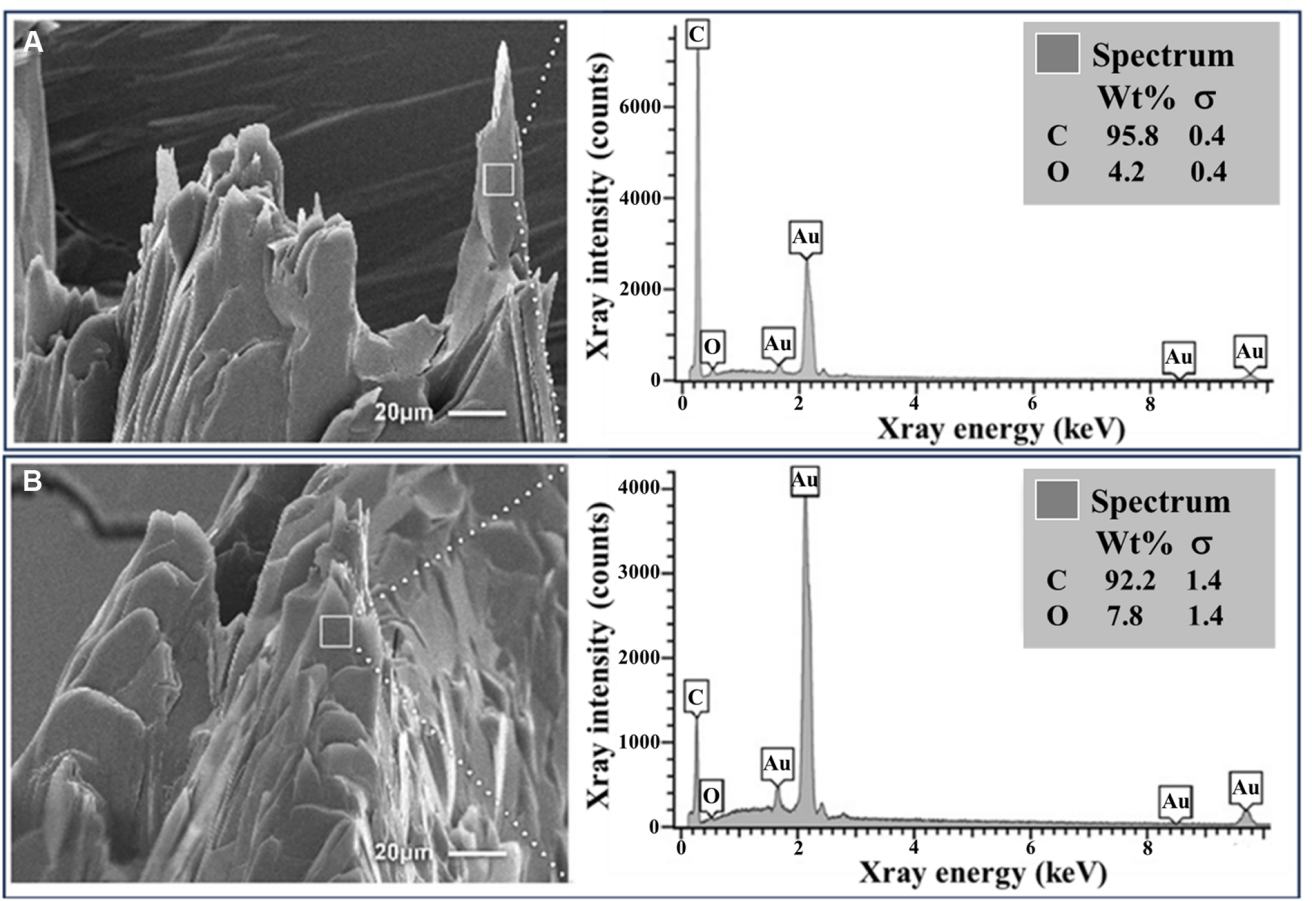
Cholesterol crystals with and without colchicine *in vitro*. (**A**) Basic cholesterol crystals with standard crystal morphology following crystallization. (**B**) Colchicine-exposed crystals with dissolving features and rounded edges. EDS demonstrates a predominance of carbon and oxygen as is consistent for cholesterol. Au, gold; C, carbon; O, oxygen.

Increasing concentration of colchicine resulted in a progressive decrease in ΔVE for the same amount of cholesterol (Figure 1). When dose response for the ΔVE with increasing dose of colchicine was evaluated with simple linear regression, the trend of decrease in ΔVE was statistically significant, *p* < 0.05 (Figure 2). The same estimate of reduction in volume expansion was observed when the linear regression model included ΔVE values for control samples in which colchicine’s value was set to 0. However, by one-way ANOVA, followed by multiple-comparisons test, only ΔVE for 5 mg/ml/g differed significantly from the control value (*p* < 0.02) (Figure 2). When Cohen's *d* effect size, which is independent of the sample size, was calculated for all pairwise comparisons of ΔVE, we observed a large effect size in all but one comparison (Table 1), indicating a significant reduction in ΔVE with colchicine. Furthermore, by SEM, cholesterol crystal morphology with colchicine modified the typical sharp tips and edges causing them to become blunted and rounded compared to control crystals (Figure 3).

**Table 1:** Effect size for decrease in ΔVE for the six pairwise colchicine dose comparisons.

Colchicine dose (mg)	*n*	ΔVE mean ± SD	ES		
			0.00	0.05	0.50
0.00	4	0.6875 ± 0.2323			
0.05	6	0.4333 ± 0.1722	0.00 vs. 0.05 = 1.29^d^		
0.50	6	0.3667 ± 0.2523	0.00 vs. 0.50 = 1.55^d^	0.05 vs. 0.50 = 0.31^b^	
5.00	6	0.1750 ± 0.1696	0.00 vs. 5.00 = 2.62^d^	0.05 vs. 5.00 = 1.51^d^	0.50 vs. 5.00 = 0.89^d^

^a^ Cohen's *d* effect size: Small, 0.2 (*n* = 0).

^b^ Cohen's *d* effect size: Medium, 0.5 (*n* = 1).

^c^ Cohen's *d* effect size: Large, 0.8 (*n* = 0).

^d^ Cohen's *d* effect size: Very large, 1.3 or greater (*n* = 5).

### 3.2 Effect of colchicine on human atherosclerotic plaques

**Figure 4:**
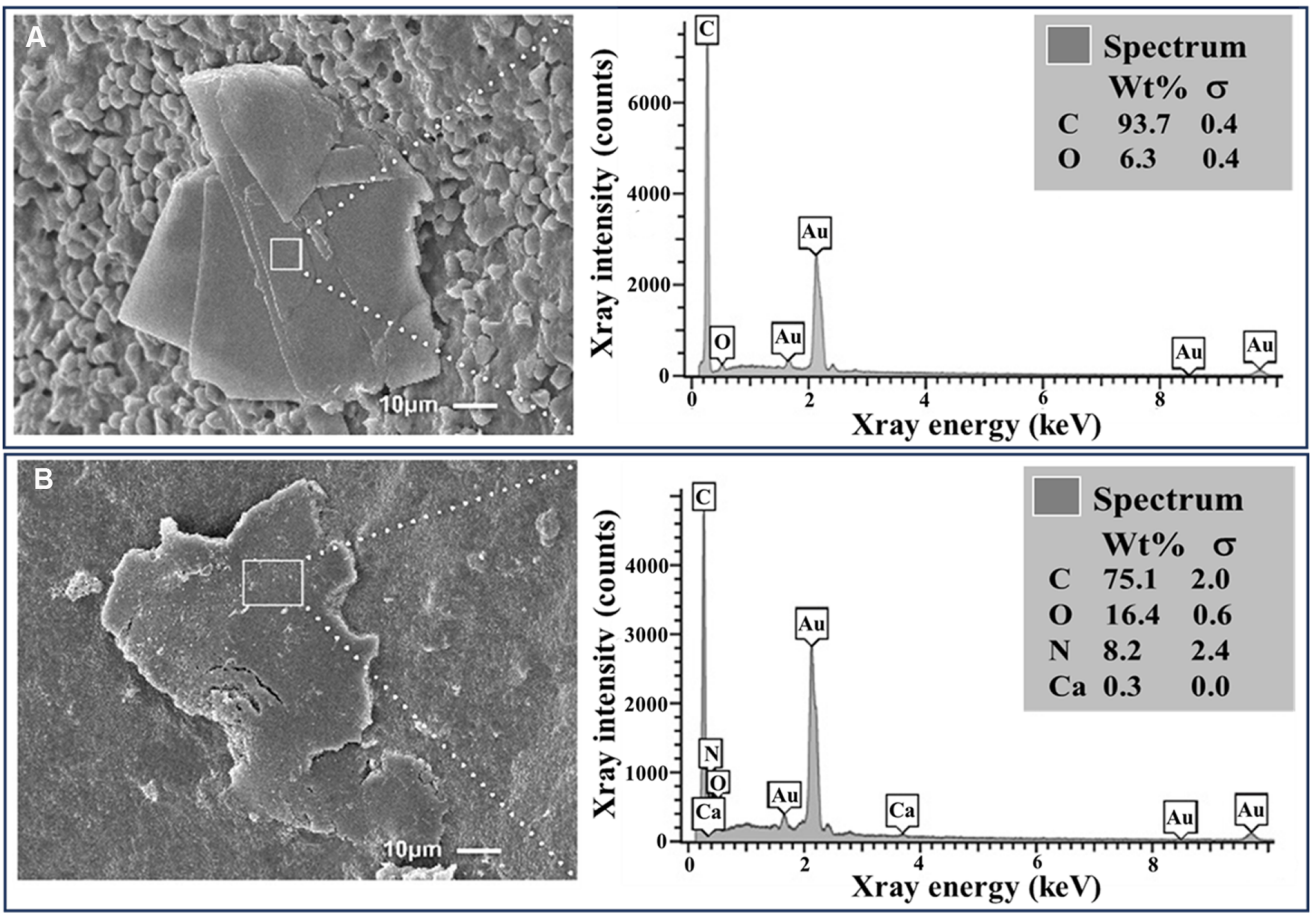
Cholesterol crystals with and without colchicine in *ex vivo* human plaques. (**A**) Intact cholesterol crystal in an atherosclerotic carotid plaque with surrounding RBCs. (**B**) Dissolving and fragmented cholesterol crystal from a plaque treated with colchicine (5 mg/ml). EDS demonstrates a predominance of carbon and oxygen with a small amount of nitrogen and calcium in the dissolving crystal (**B**) that is likely a contamination detected by the EDS from underlying tissues. Au, gold; Ca, calcium; C, carbon; N, nitrogen; O, oxygen.

SEM of the three carotid atherosclerotic plaques incubated in ddH_2_O had a similar crystal morphology as the *in vitro* controls with sharp edges but were mostly rhomboidal in shape as often seen in human plaques. However, all three colchicine-treated atherosclerotic plaque samples exhibited dissolving CCs with blunted edges and were fragmented as noted in the *in vitro* colchicine-treated samples (Figure 4). However, none of the ddH_2_O-treated samples exhibited dissolving crystals forms. Also, by visual observation, colchicine-treated samples appeared to have a lower crystal distribution compared to their matched halves incubated in ddH_2_O.

EDS showed that synthetic CCs were composed exclusively of carbon and oxygen. The CCs from the *ex vivo* carotid plaques also predominantly contained carbon and oxygen.

## 4 Discussion

This investigation evaluated the impact of colchicine on CC formation, volume expansion, and morphology in a series of bench studies that included the assessment of CCs in human atherosclerotic plaques. In the *in vitro* study, we noted a trend showing a decrease in the volume of CCs as the colchicine dose increased. The effect size was large, suggesting a practical change in volume with colchicine. The lack of statistical significance in pairwise comparisons is likely attributed to the small sample size. By SEM, CC morphology from both *in vitro* and *ex vivo* samples treated with colchicine appeared to be dissolving with the loss of sharp edges.

This research was motivated by the recognition of the pivotal role that CCs play in the development and progression of atherosclerosis and CVEs and by evidence that both statins and aspirin may also affect CC development, expansion, and morphology (11–14). By focusing on CC formation and morphology, we sought to understand if the effects of colchicine in atherosclerosis may also relate to factors other than its anti-inflammatory effects. Since colchicine is highly concentrated in leukocytes by up to 600× than serum levels, once leukocytes traverse the atherosclerotic bed, it is likely that colchicine becomes highly concentrated as senescent leukocytes become trapped in the plaque core (15, 16).

Cholesterol crystals have been shown to trigger the innate immune system via NLRP3 inflammasomes similar to uric acid crystals (2–4). Moreover, other types of crystals have been shown to cause DNA damage as in the case of silicates and asbestos that have a mutagenic potential (20). Recently, we demonstrated that CCs are also present and actively engaged in neovascularization and enhancing cancer cell tumorigenesis (21). Thus, the disruption of crystalloids that act as an irritant within the biological system could potentially inhibit the acute inflammatory response as well as the chronic irritation that is persistent causing long-term tissue injury.

Previous studies have demonstrated that statins, aspirin, and metformin, which are known to reduce cardiovascular events, attenuate CCs expansion, dissolve them, and alter their morphology while at the same time attenuating inflammation, which may reduce disease progression and the risk of CVEs (12, 14, 22–26). In contrast to colchicine, it is not known if these agents can become concentrated within the atherosclerotic bed. Cecconi et al. have demonstrated that colchicine may stabilize atherosclerotic plaque by reducing inflammatory activity and plaque burden without affecting macrophage infiltration (27). This suggests that its actions are beyond having an effect on macrophages and may be related to its effects on CC development.

Although the control test tubes demonstrate an expansion of CCs, it is also important to mention that other agents (norepinephrine and steroids) that may trigger or worsen CVE also enhanced volume expansion emphasizing that the process of inhibition is not a non-specific effect (28).

In the LoDoCo2 trial of patients with chronic coronary disease, colchicine (0.5 mg/day) was found to provide significant protection from CVE in addition to standard treatment with statins, aspirin, and beta blockers (9). Following these results, the Food and Drug Administration has approved the use of colchicine for prevention in patients who are at a high risk for CVE (17). Our study provides physiologic and morphologic effects of colchicine on CCs beyond anti-inflammation as a benefit for CVE.

## 5 Conclusion

Colchicine does affect CC formation, expansion, and morphology. This suggests that some of its clinical benefits may be due to actions beyond its well-known anti-inflammatory effects. Importantly, these results suggest that agents that specifically reduce CC formation and expansion and affect their morphology may offer a therapeutic benefit beyond that obtained with lipid-lowering and anti-inflammatory therapy.

## 6 Limitations

This study was conducted *in vitro* and *ex vivo*. An *in vivo* study would be needed to convey similar findings. However, we have conducted such a study in humans regarding statins and have shown similar CC breakdown in plaques removed from those who were on statins compared to intact CCs in those who were not taking statins (12).

## Data Availability

The original contributions presented in the study are included in the article/**Supplementary Material**, further inquiries can be directed to the corresponding author.
