# Supplementary material for: The effect of colchicine on cholesterol crystal formation, expansion and morphology: a potential mechanism in atherosclerosis
Source: Front Cardiovasc Med. 2024 Feb 16;11:1345521. doi: 10.3389/fcvm.2024.1345521 (PMC10941200; doi:10.3389/fcvm.2024.1345521)
Supplement: Supplementary file 1 [file Table1.docx]

| **Dose of Colchicine**  **(mg)** | **n** | **Mean** | **SD** | **Effect Size** | | |
| --- | --- | --- | --- | --- | --- | --- |
|  |  |  |  | 0 vs all other doses | 0.05 vs other doses | 0.5 vsother doses |
| 0.0 | 4 | 0.6875  ± 0.2323 |  |  |  |  |
| 0.05 | 6 | 0.4333  ±  0.1722 | 0.1722 | 0 vs. 0.05 =1.29 |  |  |
| 0.5 | 6 | 0.3667  ± 0.2523 | 0.2523 | 0 vs. 0.5 =1.55 | 0.05 vs. 0.5 =0.31 |  |
| 5.0 | 6 | 0.1750  ± 0.1696 | 0.1696 | 0 vs. 5 =2.62 | 0.05 vs. 5 =1.51 | 0.5 vs. 5 =0.89 |
